# Response to: ‘Body mass index and the risk of rheumatoid arthritis: a systematic review and dose–response meta-analysis’ - authors’ reply

**DOI:** 10.1186/s13075-015-0745-8

**Published:** 2015-08-19

**Authors:** Baodong Qin, Yan Liang, Zaixing Yang, Renqian Zhong

**Affiliations:** Department of Laboratory Diagnostics, Changzheng Hospital, Second Military Medical University, 415 Fengyang Road, Shanghai, China

We thank Liu and Barnabe for their valuable comments [1] on our article [2]. We think the majority of the remarks they made were reasonable and helpful for a deep understanding of the association of body mass index (BMI) and rheumatoid arthritis (RA) risk. Indeed, the three papers they mentioned discussed the role of obesity in RA development. Actually, we also identified these three papers when searching for potential papers; however, our paper is not a descriptive review, but a quantitative systematic review. We had strict inclusion criteria as described in our paper, with included studies having to fulfill the following criteria: (1) the study had a case–control or cohort design; (b) the study included risk estimates with the corresponding 95 % confidence intervals, or sufficient data for extraction or assessment; and (c) obesity, being overweight, and BMI were the exposures of interest [2]. Hemminki *et al*. [3] estimated standardized incidence ratio for RA in obese individuals compared with those who had not been hospitalized for obesity. This study was a cohort design and obesity was the exposure of interest, but we could not extract and calculate the risk estimates of RA development in the obese population. We therefore excluded this paper. Vessey *et al*. [4] reported no significant association between RA development and either weight or height. The exposure of interest was not obesity, being overweight, or BMI. Besides, no risk estimates could be obtained from this paper. For similar reasons we did not include Bartfai *et al*.'s paper [5]. Thus, we did not include these three papers due to insufficient data for calculation of pooled risk estimates.

Although several studies gave negative results, the combined results obtained from our quantitative meta-analysis have demonstrated that an increase in BMI could contribute to a higher risk for RA development. However, the real relationship between obesity, BMI and RA risk should be conducted in a large RA cohort with adjustment for more confounding factors.

## Abbreviations

BMI: Body mass index
RA: Rheumatoid arthritis
